# Chicago Medical Society

**Published:** 1889-05

**Authors:** 


### CHICAGO MEDICAL SOCIETY.

*Stated Meeting, February 18.*

THE PRESIDENT IN THE CHAIR.

Dr. N. H. PIERCE read a paper entitled

### THE BACILLUS OF KOCH AND ITS PATHOLOGICAL INFLUENCE.

It has taken practically over twenty-two years to develop the exact pathology of tuberculosis. Since 1865, when Villepin proclaimed that by vaccination with tuberculous matter an identical process like that of human tuberculosis could be produced in some of the lower animals, the contagious and infectious character of tuberculosis was partly believed or assumed by many. This, however, does not detract from the brilliancy of Robert Koch's discovery of the specific cause of tuberculosis, nor does it alter the fact that this discovery is unquestionably the most significant advance made in pathological investigation. Before Koch's memorable communication to the Berlin Physiological Society the "cheese infection theory," as explained by Buhl, and the weakly "diathesis theory" strove for supremacy as the disguise for real ignorance as to the true cause of the tubercular process.

The student who desires a plain statement of the condition of our knowledge of tuberculosis before Koch's investigations can find one in Klein's paper in the Practitioner for August, 1881. The results of Koch's investigations have been confirmed by many investigators. The result of this knowledge is used in our every day practice as data upon which we base diagnosis and treatment. The specific character of the micro-organism named by Koch, the bacillus tuberculosis, has been established.

It consists of a cell *wall* inclosing a a protoplasmic body called mycroprotein. It belongs to the smallest and finest bacillary forms known to us. Only the bacillus of mice septicæmia surpasses it in fineness. The length of the single rod ranges from one and one-half to three and one-half in.—equal to one-fourth to three-fourths the diameter of the red blood corpuscle, and their width is from one-fifth to one-sixth of their length. The ends of these rods appear to be rounded. The bacilli arising from an artificial culture fluid are in general shorter and finer than those growing in the animal organism. The largest forms are found in the sputum of phthisis.

The bacillus tuberculosis is quite able to endure the influence of any or all of the digestive secretions of the animal organism, and especially those of the stomach, as was proven by Falk in his artificial cultures and by the positive results obtained by Schell and Fischer in their feeding experiments. The tuberculosis bacillus has a very great resistance comparatively to the action of all disinfectants. Thus the tuberculosis bacillus were destroyed only after twenty hours' contact with a three per cent. carbolic acid solution. This latter substance is, therefore, not to be relied upon, especially in the disinfection of tubercular sputum, in solutions of less than five to ten per cent. Another fact of some importance is, that corrosive sublimate is not applicable in disinfection of sputum; not because of a resistance on the part of the tuberculosis bacillus to this most powerful germicide, but because it *curds* the sputum, thus materially hindering the complete mixture of the solution with all parts of the sputum. Practically we have no destructive agent that can be compared to heat, and especially moist heat, and this should be employed whereever possible.

This bacillus may gain an entrance into the human body in one of three ways: First, by respiration; second, by alimentation, and third, by inoculation. The subsequent pathological varieties will depend, first, upon the conditions of the tissues of the individual; second, the place of invasion, and the vitality or degree of malignity of the bacilli invading.

The bacillus tuberculosis never forms pus; only when the so-called tubercular granulation tissue has become infected with the staphylococcus, or streptococcus, can true pus be formed. The contents of a purely tuberculous abscess contains only a few round cells, but an amorphus shreddy material the result of the digestive power of ptomaines secreted by the bacillus tuberculosis upon the granulation tissue.

Dr. L. L. McArthur read a paper entitled

SURGICAL TUBERCULOSIS OF BONES.

In the department of osseous pathology, as in every other branch of our science, there has been made, during the last decade, such progress in our knowledge of the etiology and treatment of bone-diseases as to revolutionize the theories we have held, and offer to us in their place facts, fixed and incontrovertible, which have the impress of truth upon them, as well as the proof of actual experiment. Nor is it strange that with no exact knowledge of the ultimate causation of inflammation that theories nearly as numerous as the authorities devising them, filled our text books with vague terms to suit each writer's fancy, as well as multiply synonyms for the relatively few forms of bony inflammation; just as in the department of medicine there was a time when a diagnosis of "dropsy," "Bright's disease," or salt rheum, was esteemed sufficiently refined, though they were only results; so, until recently, we were content with an equally indefinite *result*, caries, or necrosis, which simply signifies molecular death, and death en masse. Without the slightest hint as to causation; with many theories existing there was naturally enough, great ignorance of and mystery surrounding all bone inflammations until nature solved the question for us in the result, caries or necrosis. Recently, however, there has come to us new knowledge; new facts that render clear what was obscure. Shall we permit the old ideas to be replaced by these new facts?

In considering tubercular bone disease, one or two anatomical facts have a distinct bearing on the subject in question, and on a thorough understanding of osteitis.

*First.* That the periosteum, endosteum, and medullary substance are as one, and inseparable as the peritoneum is in its various folds and turns, for the one is connected with the other by numerous blood vessels and processes, which form the union between the outer and inner membrane, as well as with the outer surface of the bone.

*Second.* That the seat of greatest vascularity and slowest circulation are in bones, the site of their diseases, and these are in cancellated or at junction of epiphyseal cartilage.

*Third.* That the nutrient artery of bones of the upper extremity run *toward the elbow*, while from the knee in the lower extremity. Now it follows that since the calcareous matter in bones only plays a passive part in any of its inflammations, there remains only its vascular skeleton, the peri and endosteum, which are only continuations of one and the same tissue, to play the active part, and since this is the source of nutrition, just so far as the function of this is interfered with just so far will we have a caries or necrosis as a result, with this difference: that whereas the products of inflammation in the periosteum can find a means of escape and thus relieve the pressure which is cutting off the nutrition of that portion of bone thus dying, inflammations of the other portion of the nourishing membrane being within an unyielding wall, any inflammation there, cuts off the nutrition of greater masses, hence gives rise to much greater constitutional disturbance, and in its efforts to escape from the confined pressure, always ends in osteitis and periosteitis.

Just here begins, from former ignorance of the cause of bone inflammation, part of the "distinction in terms without a difference," for whether it be an inflammation of the medulla, the endosteum, or periosteum it is practically an osteitis, and since there is no hard and fast boundary line between the medulla, the endosteum, and the periosteum, to make a distinction is only to confound and not to enlighten. Hence the terms endosteitis, periosteitis, and osteo-myelitis are in their pathologic nature synonymous, though differing in location. The latter is now used synonymously for all. In every bone inflammation we have two or all of three factors taking part in its production.

First. Trauma, whether from blow, cold, mineral poison or fevers.

Second. One or more of the organisms which induce suppuration.

Third. One of the organisms which induce tuberculosis, syphilis or rheumatism.

"In bony inflammations, the importance of the presence of micro-organisms can not be over-estimated," as to the casual relation they bear, while the species to which they belong determine the nature of the inflammation. When any of the organisms which induce suppuration are present, an osteo-myelitis results. When the tuberculous bacillus, syphilitic, or rheumatic organism, then an inflammation peculiar to that organism ensues. Now these things are settled, namely: That whenever micro-organisms are caught as infarcts in a tissue, they induce those pathologic changes in it which characterize that particular species.

There are often in the circulation, wholly compatible with a healthy condition, one or more of those germs whose actions on the tissues are noxious; but which without some traumatism are wholly, thanks to the leucocyte, unable to escape from their death in some of the eliminative organs, to which the blood current is hurrying them. Let, however, a lesion of any of the vessels or capillaries through which these might flow occur—a stasis even—it then becomes possible for them to wander out with or in the leucocyte, where the latter dying permits and encourages the growth of the germ in question, and all the evil consequences which this implies.

There are some peculiarities about the trauma permitting the infection. Slight traumas are frequently followed by tuberculosis; while severe injuries are not. The active reparative process in the latter case preventing the slower tuberculous process, for if there be a characteristic of bone tuberculosis that takes the precedence of all others it is its slow, and chronic nature.

Though a direct injury is a frequent element in the primary infection, the other modes of ingress are numerous.

Volckmann, the greatest living authority on tuberculosis, says that everything that heretofore has been called caries of bones, spina ventosa, pædarthrocace, belongs, with exceeding few exceptions, to tuberculosis.

Knowing the nature of tuberculosis better than before, we are now making every effort to totally and completely remove when operating the smallest suspicious focus, and as in operations for carcinoma, to repeat our work at the earliest evidence of a return. When the infective matter has been once thoroughly removed the chances of a return are not great, though it does occur sometimes in other parts—Billroth stating the proposition that if there has been an early tuberculosis, there will be a return by the age of fifty. After operations for tubercular bone disease, too, there has ensued a general miliary tuberculosis. This, however, is as rare as that other phenomena of bone surgery—fatty embolism of lungs.

It is useless to deny the spontaneous cure of tubercular osteitis when we have so many cases of spina ventosa, Pott's disease and tumor albus that have recovered without treatment. It is useless to deny the tuberculous character of an affection when a successful inoculation has been practiced.

The tubercular diathesis does not offer a just objection to operative interference.

Iodoform is the mainstay and sheet-anchor in after treatment.

